# Data Exploration and Classification of News Article Reliability: Deep Learning Study

**DOI:** 10.2196/38839

**Published:** 2022-09-22

**Authors:** Kevin Zhan, Yutong Li, Rafay Osmani, Xiaoyu Wang, Bo Cao

**Affiliations:** 1 Department of Psychiatry University of Alberta Edmonton, AB Canada; 2 Department of Cell Biology University of Alberta Edmonton, AB Canada; 3 Department of Computing Science University of Alberta Edmonton, AB Canada

**Keywords:** COVID-19, deep learning, news article reliability, false information, infodemic, ensemble model

## Abstract

**Background:**

During the ongoing COVID-19 pandemic, we are being exposed to large amounts of information each day. This “infodemic” is defined by the World Health Organization as the mass spread of misleading or false information during a pandemic. This spread of misinformation during the infodemic ultimately leads to misunderstandings of public health orders or direct opposition against public policies. Although there have been efforts to combat misinformation spread, current manual fact-checking methods are insufficient to combat the infodemic.

**Objective:**

We propose the use of natural language processing (NLP) and machine learning (ML) techniques to build a model that can be used to identify unreliable news articles online.

**Methods:**

First, we preprocessed the ReCOVery data set to obtain 2029 English news articles tagged with COVID-19 keywords from January to May 2020, which are labeled as reliable or unreliable. Data exploration was conducted to determine major differences between reliable and unreliable articles. We built an ensemble deep learning model using the body text, as well as features, such as sentiment, Empath-derived lexical categories, and readability, to classify the reliability.

**Results:**

We found that reliable news articles have a higher proportion of neutral sentiment, while unreliable articles have a higher proportion of negative sentiment. Additionally, our analysis demonstrated that reliable articles are easier to read than unreliable articles, in addition to having different lexical categories and keywords. Our new model was evaluated to achieve the following performance metrics: 0.906 area under the curve (AUC), 0.835 specificity, and 0.945 sensitivity. These values are above the baseline performance of the original ReCOVery model.

**Conclusions:**

This paper identified novel differences between reliable and unreliable news articles; moreover, the model was trained using state-of-the-art deep learning techniques. We aim to be able to use our findings to help researchers and the public audience more easily identify false information and unreliable media in their everyday lives.

## Introduction

The onset of the COVID-19 pandemic has given the world more to battle. The world has faced a barrage of false information during the “infodemic,” which is defined as the spread of a large amount of information that includes misleading or false information during a pandemic [1,2]. Due to quarantine and increased restrictions, information is trafficked to the public via social media and news sources; consequently, false information propagates at a larger scale and faster rate. Despite available public health guidelines, there is still a large presence of false and misleading information online, comprising around 20% of articles on major social media sites, such as Twitter [3]. Although the proportion of shared false information is less than evidence-informed guidelines, false information spreads at a faster rate because it contains inflammatory information [4,5]. Furthermore, infodemic management is an important aspect in maintaining public trust in scientific guidance [1]. Hence, we need to construct methods to deter the spread of false information online and identify potential sources of false news.

The abundance of fake or false news online can be instances of misinformation or disinformation and often lacks the reliability and credibility in content [6-8]. Disinformation is defined as the intentional spread of false information, while misinformation is the negligent sharing of false information [6]. Hereafter, we will not differentiate between disinformation and misinformation, as we will refer to them together as false information. False news can be categorized into 6 groups: propaganda, advertisement, manipulation, satire, parody, and fabrication [6]. Although news organizations and social media companies have implemented measures to flag and delete false news, the rate of manual false news detection is not fast enough to compete with its rapid spread through social media [9,10]. Approximately 62% of US adults obtain news from social media sites; thus, faster fact checking is critical to ensure false information spread is reduced [11]. As such, the spread of false news has resulted in public confusion, potentially associated with the antimask and vaccine rhetoric [10]. Presently, one of the most common methods to detect false news online is through human-curated fact-checking websites, such as Snopes, to flag false information [12]. Although this method may be accurate, it is inefficient due to the large amount of false news generated during the COVID-19 pandemic [10]. Thus, automatic news article reliability detection is needed.

Current false news detection using machine learning (ML) on social media has been researched extensively. Various textual features from news pages are used to predict reliability of the articles. The use of multiple features to predict the presence of false information is a common theme within current false information detection studies. The use of multiple features can improve the performance of an ML model. For example, Reis et al [13] used textual features (eg, semantic and lexical features) and news source features (eg, credibility of the news organization) as inputs for the ML model. Using traditional classifiers, such as random forest and extreme gradient boosting (XGBoost), a performance of 0.85 and 0.86 area under the curve (AUC) was achieved, respectively [13]. Elhadad et al [14] used a voting ensemble method, in addition to feature engineering, for sentiment and part-of-speech tagging. Singhania et al [15] created a 3-level HAN model using input from words, sentences, and the headline level of a news article. Similar studies have proposed that other lexical features, such as n-grams, term frequency–inverse document frequency (TF-IDF), and probabilistic context-free grammar (PCFG) have also been used as features for misinformation prediction using deep learning [16]. Accordingly, feature engineering provides higher performance metrics as well as improved interpretability. These features allow the model to focus on the important elements, which allows for reliability prediction, especially in news articles, despite high heterogeneity and noise between samples. To build on what other false information research has found, as well as to identify important new factors that contribute to false information detection, we created a final ensemble model using the ReCOVery data set [17].

Ensemble methods were implemented to further improve the performance of misinformation detection within news articles. Ensemble model usage can benefit model performance by improving the ability to generalize to data on which the model has not been trained [18]. Kumar et al [19] demonstrated improvement in performance after the use of an ensemble model, where the use of an ensemble deep learning model with a convolutional neural network (CNN) and bidirectional long short-term memory (BiLSTM) was able to achieve higher performance than a CNN or long short-term memory (LSTM) model alone, with a performance of 88.78% accuracy versus 73.29% and 80.62% for the CNN and LSTM, respectively. Due to the size of news articles, a bidirectional gated recurrent unit (BiGRU) was selected as the first model in the ensemble [20]. This model is a type of recurrent neural network (RNN) that functions well on sequential text data. A BiGRU solves the vanishing gradient problem, where the model trains on long news articles and “forgets” information from the start of the articles. This model is made of many neurons or cells, each with an update gate to control what new information is added at each word and a reset gate to control how much old information is retained. A BiGRU’s bidirectional nature allows it to process each sample from the beginning and end of the article. Compared to other state-of-the-art natural language processing (NLP) models, such as LSTM, a gated recurrent unit (GRU) has lower parameters, making it quicker to train and use [21,22]. A quicker model is important as a large number of news articles are released each day; thus, a model for false information detection needs to be both accurate and fast in order to keep up with the number of new articles. XGBoost is another model included within our ensemble model. One strength of XGboost is its exceptional ability at learning from tabular data [23,24]. As a gradient boosted tree model, it is faster than a neural network and works better on the low-dimensionality output from the first model following feature extraction. Furthermore, XGBoost has been shown to outperform deep learning models for tabular data as the hyperparameter search is shorter [24]. Additionally, XGBoost combined with deep learning models in an ensemble model yields better results than an ensemble model with multiple deep learning models or classical ML models [24].

This study aims to provide a potential solution to the multifaceted false information problem through an ensemble deep learning model to classify the reliability of news articles using the ReCOVery data set. We hypothesize that sentiment, readability, lexical categories, and other text characteristics in news articles can be used together as inputs for news reliability classification improvement. We also explore differences in the sentiment or tone of reliable and unreliable information, which can be used to classify the reliability of the text. The outcome of our study may advance news reliability classification and help researchers and the public identify unreliable news articles in their everyday lives.

## Methods

### Workflow

**Figure 1:**
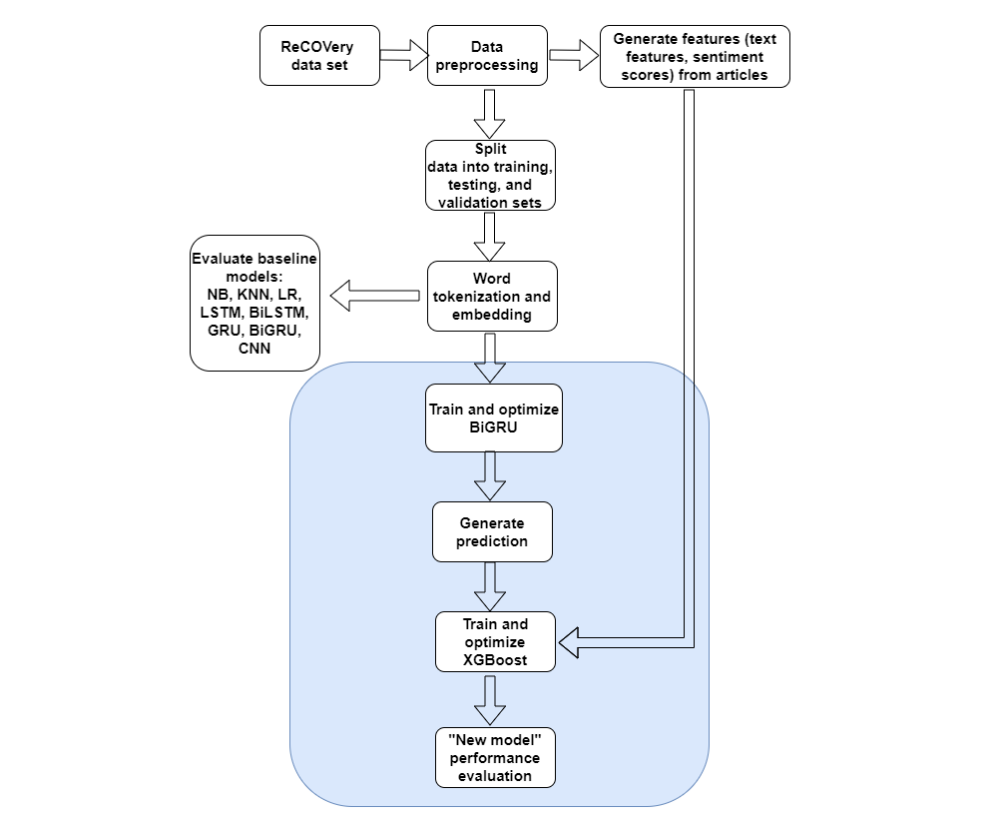
Details of workflow for data exploration and “new model” construction (highlighted in blue). CNN: convolutional neural network; BiGRU: bidirectional gated recurrent unit; BiLSTM: bidirectional long short-term memory; GRU: gated recurrent unit; KNN: K-nearest neighbor; LR: logistic regression; LSTM: long short-term memory; NB: naive Bayes; XGBoost: extreme gradient boosting.

First, data preprocessing was completed using the ReCOVery data set, which included removing stop words, links and Universal Resource Locators (URLs), and duplicate articles (Figure 1). Conversion of abbreviations and numbers to words was also completed within the preprocessing step. Following the preprocessing of the data, we performed feature engineering to create readability and sentiment scores, as well as extract lexical categories from the text (Figure 1). The preprocessed data were split into training, validation, and testing sets. Word tokenization and embedding were performed on the training and validation sets. Once tokenization and embedding were completed, 9 different ML models were trained and evaluated on the validation set to determine the best-performing model. We refer to naive Bayes (NB), K-nearest neighbors (KNNs), and logistic regression (LR) as traditional ML models as they are not deep learning models. The best-performing model was the ensemble model containing a bidirectional GRU and XGBoost ensemble “new model,” as highlighted in blue in Figure 1.

### Data Description

The ReCOVery data set was our main source of data for news articles connected to Twitter posts [17]. It focuses on the reliability of news articles from a wide array of news sources and contains 2029 articles from ~2000 different news outlets from different countries (filtered from January to May 2020) that are related to COVID-19 news [17]. Each article was labeled as either 0 for unreliable or 1 as reliable according to the NewsGuard score [17]. The NewsGuard score was developed by journalists to label the reliability of an online article. Using a scale of 0-100, the NewGuard gives points to articles that accomplish credible and transparent news practices. Online articles with a score above 60 are labeled with a “green” rating as reliable sources, and scores below 60 are labeled with a “red” rating as unreliable sources [17,25]. In addition to the NewsGuard score, ReCOVery uses Media Bias/Fact Check, which checks the correctness of news sources according to the article subjectivity and ranks articles from “very high” to “very low” in terms of factual reporting [17,26]. Reliable articles have a NewsGuard score higher than 90, with a “very high” or “high” rating on Media Bias/Fact Check. Unreliable articles have a NewsGuard score lower than 30, with a “mixed,” “low,” or “very low” factual rating on Media Bias/Fact Check [17]. The ReCOVery data set combined the NewsGuard and Media Bias/Fact Check scores to create the final news article reliability score.

### Preprocessing

Prior to data analysis, the article text and tweet data were subjected to multiple preprocessing steps. The purpose of preprocessing was to clean the data so that the deep learning model could more efficiently detect patterns in the data. The steps taken to preprocess the article text included the removal of duplicates articles or tweets; common stop words, such as “the” and “a”; and all links and non-English characters. Lemmatization of the article text was also completed, in addition to the conversion of acronyms to full terms.

Preprocessing was conducted using Python libraries, such as Pandas and Natural Language Toolkit [27,28]. A total of 1346 reliable articles and 648 unreliable articles were used for model training. Additionally, 34 articles were removed as they had less than 100 words, which limited the validity of reliability analysis. Following preprocessing, features from the news articles such as text characteristics, readability, and sentiment were extracted for analysis and to be included as input to the deep learning model.

### Sentiment Analysis

Sentiment analysis was applied to the body text of reliable and unreliable articles. This was implemented through Valence Aware Dictionary and Sentiment Reasoner (VADER) and TextBlob, which are open source tools for determining predominant sentiment, polarity, and subjectivity [29,30]. The analysis relies on lexicographic analysis to map the text features of each article to different scores with regard to sentiment, polarity, and intensity. In terms of sentiment, the articles have a continuous score between 0 and 1, including both endpoints, with 1 representing that the article contains the specified sentiment as the predominant sentiment. For example, if an article has a positive sentiment of 1, this means the article contains the highest-possible positive sentiment. VADER and TextBlob were imported into Python and applied to the body text of articles within the data set. The total proportion of articles with a positive, negative, and neutral sentiment were determined through library functions within VADER and TextBlob.

### Text Analysis

**Figure 2:**
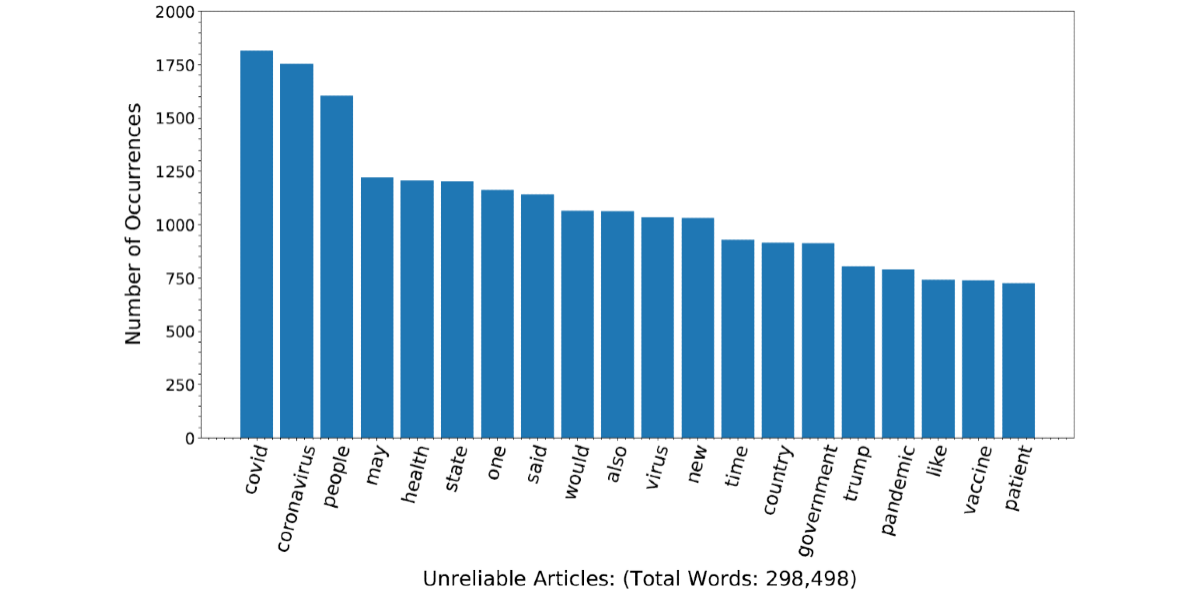
Number of occurrences for keywords in unreliable news articles (N=298,498 words).

**Figure 3:**
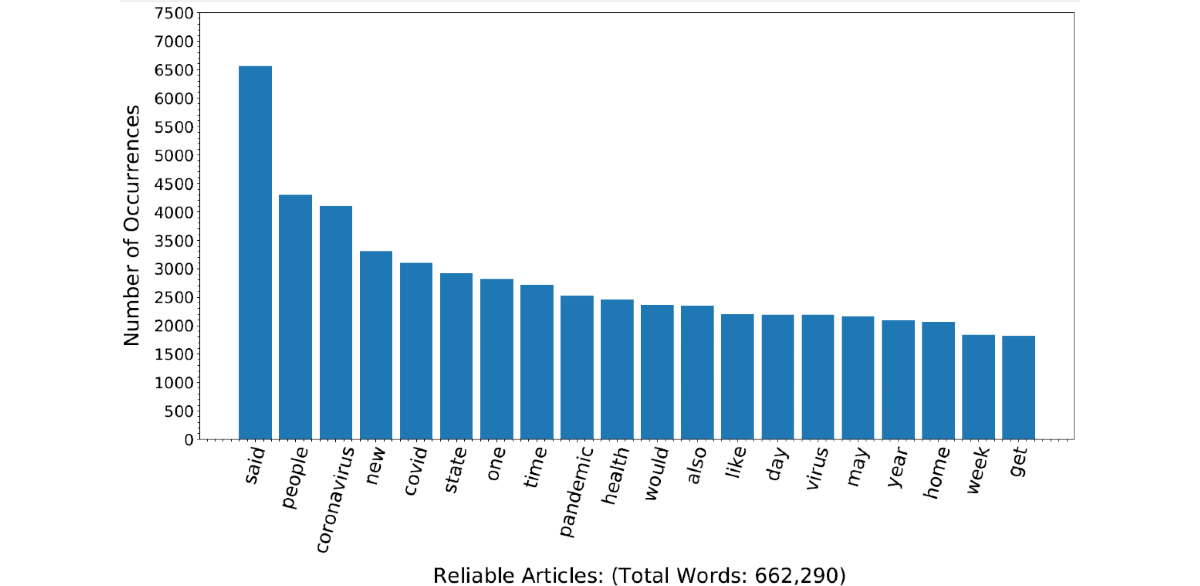
Number of occurrences of keywords in reliable news articles (N=662,290 words).

After preprocessing, the body text of articles was analyzed. The most common words from reliable and unreliable articles were determined. They are presented in a frequency bar graph to demonstrate the major differences between unreliable and reliable articles (Figures 2 and 3, respectively). Another feature included within the deep learning model was the text length and readability of the newspaper articles. The length of the articles was assessed using the character length of the article sentences and overall article length. Readability was assessed using 6 different readability metrics from the py-readability-metrics library: the Flesch-Kincaid grade level, Gunning fog index, Coleman-Liau index, Dale-Chall index, automated readability index (ARI), and Linsear Write index [31]. The aforementioned readability metrics are used to determine the grade level necessary to understand a written document based on the sentence length and word length [32].

The Flesch-Kincaid grade level is a scale modified from the Flesch-Kincaid reading ease index that compares the ratio of words per sentence and the ratio of syllables per word [33]. The values for this scale linearly indicate the estimated US grade level of a text. For example, a grade of 10-12 would indicate that the target reader is at the high school level, whereas scores higher than 12 are graduate-level texts [33]. Similarly, the Coleman-Liau index and the ARI both assess character and word frequency to approximate the US grade level required to read a text [34]. The Gunning fog index assesses the frequency of difficult words in a text and is a linear range between 0 and 20: a score of 16-20 is at the graduate level [35]. Similarly, the Dale-Chall index evaluates the frequency of difficult words but is scaled so that a score of 9-10 represents a university graduate–level text [31,36-38]. Lastly, the Linsear Write index was developed to assess the readability of technical texts, and its score represents the years of formal US education needed to understand a text, similar to the previous indices [39].

Topic analysis was performed using Empath, a neural network–based lexicon [40]. Empath is able to determine whether a certain sentence has the lexical categories of politics, religion, contentment, and approximately 200 more categories [40]. By processing the text with Empath, we derived 194 lexical categories that were used as additional features that were concatenated with the previous text, sentiment, and readability features in the final deep learning model. The extracted lexical categories from Empath increased the amount of information the deep learning model trained on for each article and allowed for better interpretability as differences in topic frequencies could also be evaluated. For each of the lexical categories, a mean count for reliable and unreliable articles was derived, along with the *t* test and the *P* value (Table 1).

**Table 1:** Top 10 lexical categories from Empath (a neural network–based topic analysis tool) in reliable and unreliable news articles selected by Empath. The reliable and unreliable means is the mean counts of each lexical category being classified into reliable and unreliable news articles, respectively.

Lexical category	*t* (*df*)	*P* value	Reliable mean (SD)	Unreliable mean (SD)
magic	–7.91 (1992)	<.001	0.19 (0.60)	0.51 (1.22)
power	–7.16 (1992)	<.001	1.28 (2.20)	2.16 (3.24)
business	7.15 (1992)	<.001	8.58 (10.54)	5.31 (7.10)
work	6.89 (1992)	<.001	5.78 (8.82)	3.28 (3.89)
contentment	6.18 (1992)	<.001	0.70 (1.61)	0.29 (0.72)
office	6.14 (1992)	<.001	3.02 (4.37)	1.88 (2.60)
dispute	–6.11 (1992)	<.001	1.58 (2.48)	2.35 (2.94)
morning	5.87 (1992)	<.001	1.06 (1.87)	0.59 (1.11)
legend	–5.85 (1992)	<.001	0.34 (0.92)	0.64 (1.31)
blue collar job	5.83 (1992)	<.001	0.62 (1.75)	0.21 (0.68)

### Tokenization

As ML models only accept numerical inputs, the text data must be tokenized. This process involves a word-index dictionary, where each word in the data set is converted to a numerical value or index, which corresponds to that word in the dictionary. For example, a word such as “coronavirus” might be presented to a ML model as the value 1234. As each unique word creates a unique index number, the “vocabulary” or total number of unique words in the data set can be a problem, especially if the data set is large, since words that appear once or twice in the data set generally do not contribute to the efficacy of the model. We limited the vocabulary size to 20,000 (51.73%) out of a total of 38,663 unique words from the training data. This excluded words that were used only once in the data set, because these words would not be useful to the model—Zipf’s law reaffirms that having larger vocabulary sizes gives diminishing returns as we frequently use a small proportion of their total vocabulary [41,42]. Furthermore, there are various estimates regarding the vocabulary size of an average native English speaker, with around 20,000 being a reasonable estimate for the vocabulary size [43,44]. Articles were also 0-padded to a size of 3500 words, which was the size of the longest article to ensure uniformity of the model input.

### Word Embedding

Following tokenization, the data were processed using word embedding, a form of unsupervised ML. Word embedding places the data points of individual words into an embedding space with high dimensionality. Inside this embedding space, each word is represented as a vector with words that are similar to each other being located in close proximity. As such, word embedding allows hidden relationships between similar words to be quantified for ML analysis. Although a new word embedding layer could be trained and fitted on our data set, there exist pretrained word embedding models that are more efficient to use. For the article text data, we leveraged Global Vectors for Word Representation (GloVE), which is a commonly used word embedding model trained on hundreds of thousands of Wikipedia articles, which have an embedding space of 100 dimensions [45].

### Machine Learning Classification

The data were randomly split into training, testing, and validation subsets for deep learning. The ratio of these subsets was 8:1:1, respectively. Of the 1994 articles, 1595 (79.99%) were in the training subset, 199 (9.98%) were in the validation subset, and 200 (10.03%) were in the testing subset. The training and validation data were used to build the model to classify between reliable and unreliable articles, while the testing data were used to evaluate the model’s performance. The splitting of the data followed by model training and evaluation were repeated 10-folds so that each article could be included in the training set. An average was taken between the performance metrics obtained from training on each fold. We evaluated the performance of multiple ML models on the data set (NB, KNNs, LR, LSTM, GRU, BiLSTM, BiGRU, and CNN) to determine the best models for reliability detection. The settings or hyperparameters were optimized either experimentally or using Gridsearch, which tests all combinations of hyperparameters for each of the aforementioned ML models.

Finally, we developed an ensemble model using a lightly trained BiGRU to generate an initial reliability prediction, which was then combined with the text features, readability, sentiment, and Empath-classified lexical categories. This was then used to train an XGBoost model with 10-fold cross-validation.

This paper uses several evaluation metrics that rely on the results from the confusion matrix. These metrics were derived from correct predictions by the model, such as true positive (TP) and true negative (TN), as well as incorrect predictions, such as false positive (FP) and false negative (FN). Accuracy is the total proportion of correct predictions, but this evaluation metric is not as effective when there is a class imbalance. Sensitivity refers to the proportion of correctly predicted unreliable articles, while specificity refers to the proportion of correctly predicted reliable articles. The AUC score shows the performance of the model at different TP and FP rates [46].


Sensitivity (recall) = TP/(TP + FN)

Specificity = TN/(TN + FP)

Accuracy = (TP + TN)/(TP + TN + FP + FN)

### Ethical Considerations

The data used in this paper did not need ethics approval as they were accessed through the open access ReCOVery data set GitHub, as cited in Zhou et al [17].

## Results

### Data Exploration

Data exploration was performed and features, such as readability, sentiment, and lexical categories, were combined with the full news article text data to train an ensemble model. An ensemble method using BiGRU and XGBoost was created using 1346 reliable articles and 648 unreliable articles.

During data exploration, we found that the average text length in terms of the average word length and sentence length was longer in unreliable articles compared to reliable articles (Table 2). The Flesch-Kincaid grade level, the Dale-Chall index, the ARI, the Coleman-Liau index, the Gunning fog index, and the Linsear Write index indicated that reliable articles are easier to read compared to unreliable articles (Table 2). From the average frequency of 194 Empath-derived lexical categories, 110 (56.7%) were significantly different between reliable and unreliable articles (Multimedia Appendix 1). Most frequent words in unreliable and reliable articles were also visualized (Figures 2 and 3, respectively). Unreliable articles had higher rates of negative sentiment, while reliable articles had higher rates of neutral sentiment (Table 3). Performance metrics of various trained ML models as well as the new ensemble model were determined (Table 3).

**Table 2:** Text length and readability metrics for reliable (N=1346) and unreliable (N=648) online news articles. The text length was expressed as the average sentence length and word length. Readability was expressed using the Flesch-Kincaid grade level, the Dale-Chall readability index, the ARI^a^, the Coleman-Liau index, the Gunning fog index, and the Linsear Write index.

Metrics	Reliable mean (SD)	Unreliable mean (SD)	*t* (*df*)	*P* value
Average word length (characters)	6.14 (0.27)	6.32 (1.66)	–3.93 (1992)	<.001
Average sentence length (words)	23.67 (5.17)	26.38 (7.06)	–9.70 (1992)	<.001
Flesch-Kincaid grade level	12.68 (2.63)	14.39 (3.37)	–12.38 (1992)	<.001
Gunning fog index	14.87 (2.72)	16.42 (3.33)	–11.00 (1992)	<.001
Coleman-Liau index	10.85 (1.87)	11.82 (2.46)	–9.72 (1992)	<.001
Dale-Chall index	10.21 (0.96)	10.70 (1.02)	–10.53 (1992)	<.001
ARI	13.41 (3.30)	15.43 (4.47)	–11.41 (1992)	<.001
Linsear Write index	16.42 (4.02)	18.73 (5.31)	–10.80 (1992)	<.001

^a^ARI: automated readability index.

**Table 3:** Comparison of sentiment polarity (0=least expression of sentiment in interest, 1=most expression of sentiment in interest) between reliable (N=1346) and unreliable (N=648) news articles in terms of sentiment of the sentences within news articles. Differences between the frequencies of sentences possessing positive, neutral, or negative sentiment were analyzed with a 2-sample independent *t* test.

Sentiment	Reliable mean (SD)	Unreliable mean (SD)	*t* (*df*)	*P* value
Negative	0.066 (0.042)	0.076 (0.039)	–5.46 (1992)	<.001
Neutral	0.850 (0.054)	0.840 (0.050)	4.37 (1992)	<.001
Positive	0.084 (0.035)	0.085 (0.035)	–0.095 (1992)	.92

### Text Analysis

After removal of stop words, the most frequent words in reliable and unreliable articles were examined. The highest word frequencies for unreliable and reliable articles are illustrated in frequency bar graphs (Figures 2 and 3). Common words between reliable and unreliable news articles were COVID-19–related keywords, such as “coronavirus,” “virus,” and “pandemic.” The differences were related to political undertones, such as “Trump” and “government.” Additionally, the Empath lexicon tool was applied to the text to yield lexical categories. The average count for each lexical category was determined for reliable and unreliable text. The differences in means were then compared using *t* tests. There were a total of 194 lexical categories that significantly differed in frequency between reliable and unreliable texts (Multimedia Appendix 1 and Table 1). In Table 1, we display the top 10 lexical categories with the lowest *P* value. Categories included “magic,” “power,” “business,” “work,” “contentment,” “office,” “dispute,” “morning,” “legend,” and “blue collar job.” The lexical categories “business,” “work,” “contentment,” “office,” “morning,” and “blue collar job” had higher mean counts for the reliable articles compared to the unreliable articles. The lexical categories “magic,” “power,” “legend,” and “dispute” had lower mean counts for the reliable articles compared to the unreliable articles. In terms of text characteristics, there was a significant difference in the average sentence length between reliable and unreliable news articles, with reliable articles containing shorter sentences at 23.67 (SD 5.17) words per sentence compared to unreliable articles containing 26.38 (SD 7.06) words per sentence (Table 2). Additionally, the average word lengths were 6.14 (SD 0.27) and 6.32 (SD 1.66) for reliable and unreliable articles, respectively. In addition to text length, we also analyzed the differences in readability between reliable and unreliable articles. The readability indices used were the Flesch-Kincaid grade level, the Dale-Chall index, the ARI, the Coleman-Liau index, the Gunning fog index, and the Linsear Write index. As shown in Table 2, unreliable articles were less readable, as indicated by all 6 readability indices. Since these text features are important in differentiating between reliable and unreliable news articles, they were input into our final deep learning model.

### Sentiment Analysis

Using VADER, the sentences from the articles were classified into positive, neutral, and negative sentiments. The sentiment score ranged from 0 to 1, with 1 denoting strong presentation of the sentiment of interest. For reliable articles, the means for the negative, neutral, and positive sentiments scores were 0.066 (SD 0.042), 0.850 (SD 0.054), and 0.084 (SD 0.035), respectively (Table 3). For unreliable articles, the means for the negative, neutral, and positive sentiment scores were 0.076 (SD 0.039), 0.840 (SD 0.050), and 0.084 (SD 0.035), respectively.

### Machine Learning Analysis

**Figure 4:**
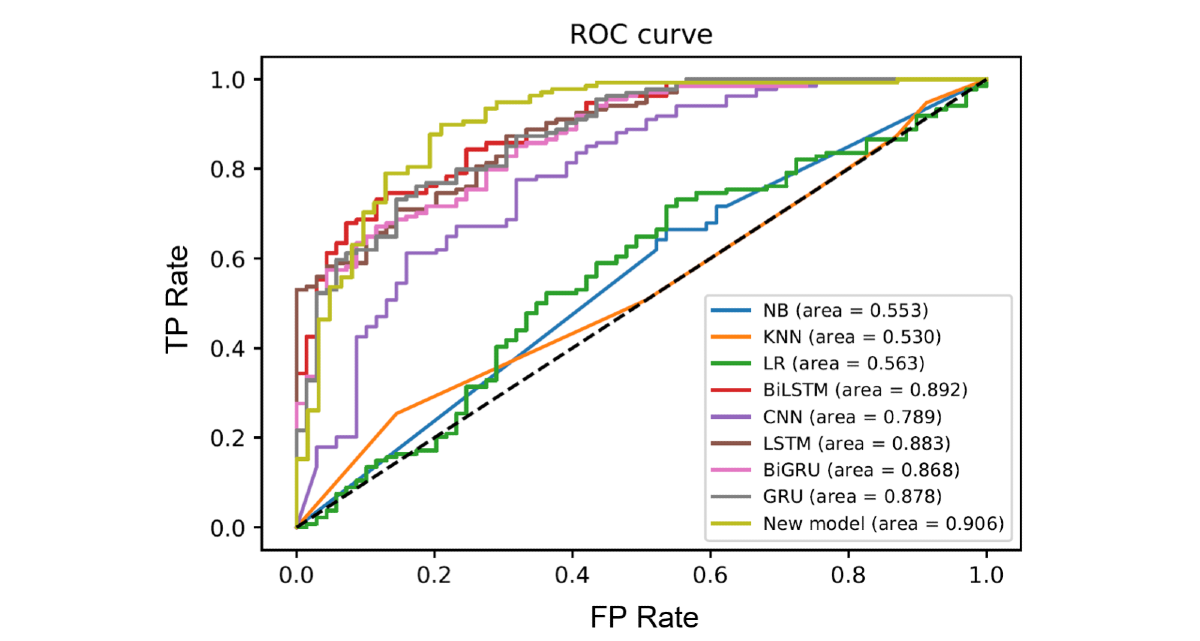
Receiver operating characteristic (ROC) curve and AUC scores with the corresponding color for both traditional ML models (KNN, LR,NB) and deep learning models (BiLSTM, CNN, LSTM, BiGRU, GRU, new model). AUC: area under the curve; BiGRU: bidirectional gated recurrent unit; BiLSTM: bidirectional long short-term memory; CNN: convolutional neural network; FP: false positive; GRU: gated recurrent unit; KNN: K-nearest neighbor; LR: logistic regression; LSTM: long short-term memory; ML: machine learning; NB: naive Bayes; TP: true positive.

After the newspaper article data were passed through GloVE word embedding, the text data were split 10-folds for cross-validation. The traditional ML models included LR, KNNs, and NB. The AUC values (Figure 4) were generated, in addition to sensitivity and recall values (Table 4).

Next, the deep learning models were fit to the data. Each model included the GloVE word embedding prior to training. Optimization of hyperparameters for the deep learning models was completed using GridSearchCV from the ML Python scikit-learn library. The hyperparameters optimized were batch size, epochs, dropout rate, neuron number, optimizer type, learning rate, and activation function type. Each model had varying hyperparameters that yielded the best results.

The deep learning models that were assessed were LSTM, GRU, BiLSTM, BiGRU, and CNN. Similar to traditional ML models, the AUC, specificity, and recall were determined as performance metrics (Table 4).

**Figure 5:**
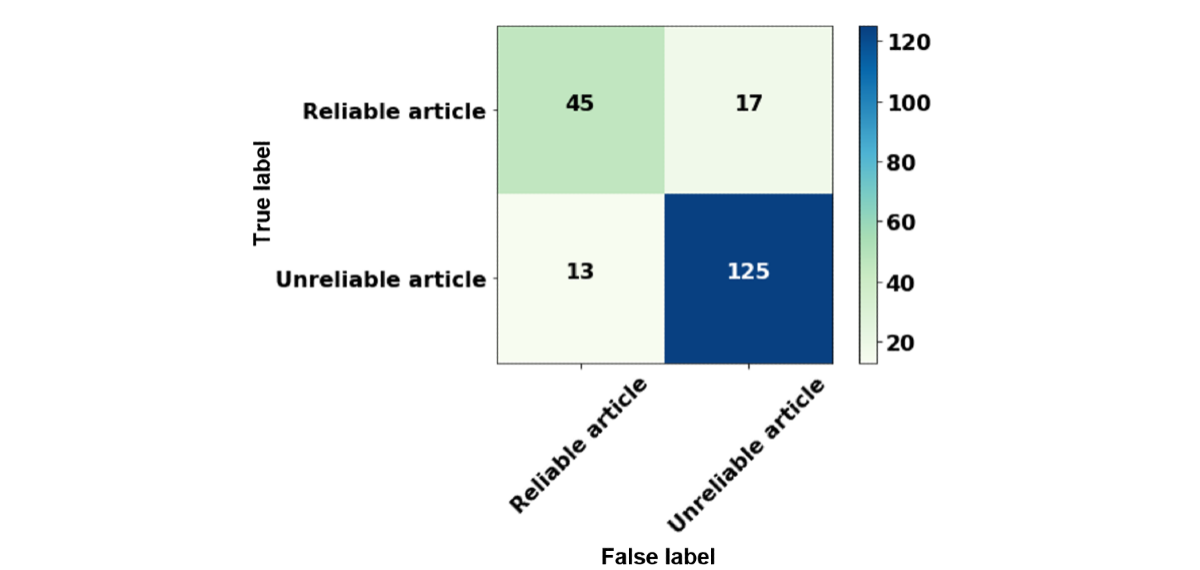
Confusion matrix for ReCOVery validation subset on trained new ensemble model with BiGRU and XGBoost. BiGRU: bidirectional gated recurrent unit; XGBoost: extreme gradient boosting.

Lastly, an ensemble model was developed using the BiGRU and XGBoost. Our new model was first evaluated on the ReCOVery testing subset. A confusion matrix for our new model was generated, as shown in Figure 5. The AUC, specificity, and sensitivity for our new deep learning model were 0.906, 0.835, and 0.945, respectively (Table 4).

**Table 4:** Performance metrics for the ReCOVery validation data set for traditional ML^a^ models (KNN^b^, LR^c^, NB^d^), and deep learning models (BiLSTM^e^, CNN^f^, LSTM^g^, BiGRU^h^, GRU^i^, new model).

Model	Specificity	Sensitivity	AUC^j^
LR	0.720	0.575	0.563
KNN	0.660	0.739	0.530
NB	0.700	0.627	0.553
BiLSTM	0.810	0.925	0.892
CNN	0.792	0.851	0.789
LSTM	0.829	0.903	0.883
BiGRU	0.791	0.963	0.868
GRU	0.804	0.918	0.878
New model	0.835	0.945	0.906

^a^ML: machine learning.

^b^KNN: K-nearest neighbor.

^c^LR: logistic regression.

^d^NB: naive Bayes.

^e^BiLSTM: bidirectional long short-term memory.

^f^CNN: convolutional neural network.

^g^LSTM: long short-term memory.

^h^BiGRU: bidirectional gated recurrent unit.

^i^GRU: gated recurrent unit.

^j^AUC: area under the curve.

## Discussion

### Principal Findings

This study demonstrates an ensemble model with BiGRU and XGBoost for text reliability classification using the ReCOVery data set with a specificity, sensitivity, and AUC of 0.835, 0.945, and 0.906, respectively [17]. Through our data analysis, we demonstrated that unreliable news articles have lower readability and higher sentence length. They also include more negative and less neutral sentiments and contain more polarizing lexical categories in comparison to reliable articles.

### Data Usage

With regard to using news articles to build a classification model, an important consideration is the generalizability of the model. To ensure that the model is generalizable, the data used to train the model must be diverse in nature. A shortcoming of many deep learning misinformation detection studies is the focus on detecting misinformation from a narrow range of news sources, or locations [17,47]. Because of the homogenous nature of the data set used to train these models, many misinformation detection models are potentially less generalizable [47]. An example would be CoAID, a data set constructed from COVID-19–related news articles and social media posts from December 1, 2019, to September 1, 2020. A shortcoming of the CoAID data set would be the lower number of news sources used for the data set as 9 reliable news sources were included during the data collection process [48]. CoVerifi is a study that used the CoAID data set to create a web-based tool to check whether an online news article was credible [49]. Another notable data set is the COVID-19-FAKES data set containing 61,711 tweets with misinformation and 2,985,399 tweets without misinformation [50,51]. Silva et al [51] used the COVID-19-FAKES data set to obtain insights into predictive features for the presence of misinformation in tweets and the differential engagement in tweets with and without misinformation [51]. Hence, we used the ReCOVery data set for the diverse nature of the news articles as they range from ~2000 different news outlets from different countries [17].

### Sentiment Analysis

VADER was used to evaluate sentiment at a lexicon-based level due to its high accuracy, with an *F*1 classification accuracy of 0.96 and computational economy [29]. Although VADER has become a staple in NLP for sentiment analysis, 2 key shortcomings to consider are its inability to recognize sarcasm/satire and its reduced accuracy when dealing with 3-class analyses (negative, neutral, and positive) [52].

From the distribution of articles with reliable versus unreliable news articles, it can be observed that reliable articles contain less negative sentiment in comparison to unreliable articles as they had a lower negative sentiment polarity score (Table 3). This is in line with observations of news content in the literature, as Arif et al [53] discussed how individuals searching for negative terms on the internet can lead to more biased articles. To emphasize the importance of sentiment in differentiating fake and real news, Paschen [54] concluded that the titles and body text of fake news articles contain more negative content, such as anger and disgust, compared to real news articles. Fake news is more likely to display negative sentiment to drive a specific narrative for profit, which supports our finding that there are a greater number of negative unreliable sources than neutral or positive unreliable sources.

We observed a difference between the number of neutral reliable and neutral unreliable articles, with more neutral sentiment in reliable articles in comparison to unreliable articles (Table 3). A neutral sentiment scoring for reliable data sources implies impartiality and objectivity when discussing the subject matter [55].

Many ML studies have targeted sentiment as a feature to predict misinformation in a variety of written information online because of the different sentiment valence between reliable and unreliable text due to the aforementioned reasons [56]. Because of the differing nature of sentiment between texts of differing reliability, sentiment analysis was used in the context of filtering out negative messages on social media, spam filtering, among other applications [56]. In agreement with our findings, Ajao et al [57] determined that unreliable tweets often contain more negative sentiment in comparison to reliable tweets due to how authors of unreliable tweets use negative emotions to better propagate their message. They also showed that the use of sentiment can boost support vector machine (SVM) accuracy when the sentiment is considered in addition to textual features [57]. Hence, sentiment was a feature selected for our model.

### Text Analysis

The words themselves were observed to be quite similar to one another between the 2 groups because the subject matter of both reliable and unreliable sources is the same: COVID-19. Additionally, many of the most frequently occurring words are mere transitional words that are likely to be found in the majority of English literature.

Interestingly, the most frequently occurring word in reliable sources was “said” (Figure 3). This is likely due to “said” being used to quote political figures and leaders in the scientific field. The reliability of articles in this case is a consequence of the articles citing reliable sources of information. Another observable trend is the increasing number of politically charged words found in unreliable articles. Words such as “country,” government,” and “Trump” were amongst the most frequent words for unreliable sources but not for reliable articles (Figure 2). This communicates a pattern of political commentary occurring in unreliable sources [58]. We can anticipate that articles discussing political content in the context of COVID-19 are likely interested in propagating an agenda—hence, the unreliability. For example, Chen et al [59] found interplay between COVID-19 misinformation propagation and the 2020 US presidential elections with regard to mask use and mail-in ballots. Specifically, health information has been politicized to push political agendas and attack political opponents. In addition to frequently occurring words, lexical categories extracted from Empath and similar models allows us to evaluate the difference in topic frequencies between reliable and unreliable news articles [40]. The use of lexical categories extracted from Empath and similar models can increase model performance compared to using only raw text data [60-63].

Another feature we decided to explore and include in our final deep learning model is the readability and length of the news articles. Readability has been shown to be predictive of misinformation. In the study by Santos et al [64], articles from a frequent source of fake news could be differentiated using only article readability scores with an SVM algorithm with an accuracy of 92% [64]. Similarly, in a study by Zhou et al [65], various metrics were explored based on their ability to classify reliable versus unreliable news articles. It was determined using random forests that readability is among the top 5 in terms of contribution to the model, alongside sentiment [65].

### Machine Learning Classification

In the original ReCOVery study, Zhou et al [17] created a baseline prediction performance for news article reliability and found that a precision of 0.721-0.836 and 0.421-0.667 can be obtained for reliable and unreliable news articles, respectively. A recall of 0.705-0.829 and 0.441-0.667 can be obtained for reliable and unreliable news articles, respectively [17]. The features used in the baseline model ranged from text lexical categories, rhetorical structure, and visual information within news articles. Zhou et al [17] also tested the model on traditional ML models, such as SVMs, or deep learning algorithms, such as CNNs with unimodal and multimodal features. Other studies have also explored the use of the ReCOVery data set for false information classification. One such study is by Raj and Meel [66], where a novel deep learning model, the Allied Recurrent and Convolutional Neural Network (ARCNN), was created using both image and textual features within news articles to detect misinformation. The performance of the ARCNN was tested using 6 COVID-19 fake news data sets, with ReCOVery as 1 of the data sets, achieving an accuracy, precision, recall, and *F*1 score of 80.98%, 53.85%, 58.33%, and 56.00%, respectively [66]. Another study using the ReCOVery data set for model development explored the use of multiple languages for fake news detection to improve model performance [67]. Finally, Wahle et al [68] used the ReCOVery data set as 1 of 6 COVID-19 misinformation data sets to evaluate the performance of 15 transformer-based ML models to determine the generalizability of different transformer models. Differing from the aforementioned studies, we were able to demonstrate that the use of readability, text characteristics, sentiment, and lexical categories can improve upon the original ReCOVery data set baseline models [17]. Hence, we demonstrate the importance of the aforementioned text features to improve upon news article reliability prediction. Furthermore, we show that the combination of multiple inputs and consideration of the chosen model can increase ML model accuracy in the context of NLP.

In our final proposed model, the BiGRU with XGBoost and feature engineering was the best-performing model. A BiGRU is adept at capturing temporal data in long sequences, as bidirectional models can better capture the context of the text [46]. During the experimentation with these models on ReCOVery data, we found that all deep learning models outperformed the traditional ML models because deep learning models are better able to handle more complex data [46,69]. Furthermore, we chose to use the GRU algorithm, which is a variant of the recurrent neural network, in addition to the LSTM algorithm due to the increased performance on longer text compared to LSTM [21]. To further increase the performance of our model, an ensemble model was built, as combining multiple predictions can yield more accurate predictions [70].

### Strengths

A strength of our investigation is that it not only had the main goal of creating a deep learning model for reliability prediction but also identified significant trends in text and sentiment for reliable and unreliable news articles. An investigation focused solely on a deep learning model has a “black box” problem where the mechanisms used by the deep learning model are not visible and are contained within its many complex hidden layers [71]. As such, a data exploration approach coupled with the deep learning model is able to better visualize and portray article reliability classification. Furthermore, our paper examined news articles, which had the advantage of being more normalized in text compared to tweets and social media as, each article was written with a professional approach. As such, less data were removed during preprocessing due to grammatical or spelling errors. Using news articles as data also avoided the problem of low hydration that Twitter misinformation data sets suffer from when tweets are removed by Twitter.

### Limitations and Future Directions

There are a number of ways our project could be further refined. First, expanding the number of total available data would be valuable as there are nearly twice as much data for reliable sources as unreliable. Furthermore, being able to web-scrape Facebook postings and Reddit threads would allow us to expand our scope of access and evaluate other high-traffic sources of information. Incorporating clustering models would also increase the specificity of our search and create a more accurate model that can consider what aspect of COVID-19 is being discussed when determining reliability. Due to the high accuracy of our model, as shown by the results, our model can be commercialized as a web app that allows users to assess, to a high degree of confidence, the reliability of the article they are reading. Moreover, it can also be used to determine the sentiment scoring of an article to determine whether they want to engage in that specific literature.

Although this model specifically identifies COVID-19–related information, it could also be trained for other types of misinformation. As discussed previously, most current methods to combat misinformation online are through the use of human-moderated fact-checking websites. Examples include Twitter's Birdwatch program, where independent users can flag posts they deem untrustworthy [72]. Other methods used include Facebook's fact-checking service, which manually labels posts or websites containing misinformation as untrustworthy and removes them from public view [73]. Furthermore, warnings are placed below posts containing COVID-19 information to warn readers regarding potential misinformation contained within posts [73]. Even though there are numerous instances of fact checking, the major issue that arises is the inefficiency in manual fact checking [74]. Hence, new fact-checking methods aim toward automating the fact-checking process. The first example of a fact-checking website is the Bot Sentinel automated Twitter fact-checking software, which can be installed by users to monitor spam accounts [75]. Bot Sentinel uses ML technology to classify posts or profiles as reliable or unreliable to an accuracy of 95% [75].

### Conclusion

In conclusion, we demonstrated that readability, sentiment, text characteristics, and lexical categories are important in differentiating between reliable and unreliable news articles, as it was shown that unreliable articles are less readable, have more negative sentiment, and have more political lexical categories. The aforementioned features were used to achieve above-the-baseline performance within the original ReCOVery data set, with a specificity, sensitivity, and AUC of 0.835, 0.945, and 0.906, respectively, using our new ensemble deep learning model. Hence, the application of readability, sentiment, and lexical categories using our new model can help determine the dependability of news articles and better improve upon pre-existing models that do not use these features.

COVID-19 has brought to light the importance of developing an automated reliability assessor for news articles, as human-moderated fact-checking methods may be inefficient. Because readability, sentiment, and lexical categories can be used to improve upon pre-existing reliability classification models, we show that automated reliability detection may be an alternate way to determine new article reliability in the future, which will help news readers identify articles containing potentially unreliable information.

## Appendix

Multimedia Appendix 1 Mean (SDs) scores for Empath categories of reliable and unreliable news articles.

## Abbreviations

ARCNN: Allied Recurrent and Convolutional Neural Network
ARI: automated readability index
AUC: area under the curve
BiGRU: bidirectional gated recurrent unit
BiLSTM: bidirectional long short-term memory
CNN: convolutional neural network
FN: false negative
FP: false positive
GloVE: Global Vectors for Word Representation
GRU: gated recurrent unit
KNN: K-nearest neighbor
LR: logistic regression
LSTM: long short-term memory
ML: machine learning
NB: naive Bayes
NLP: natural language processing
SVM: support vector machine
TN: true negative
TP: true positive
VADER: Valence Aware Dictionary and sEntiment Reasoner
XGBoost: extreme gradient boosting
